# Trends in neonatal mortality in Nigeria and effects of bio-demographic and maternal characteristics

**DOI:** 10.1186/s12887-015-0349-0

**Published:** 2015-04-09

**Authors:** Joshua Odunayo Akinyemi, Elijah Afolabi Bamgboye, Olusola Ayeni

**Affiliations:** Department of Epidemiology and Medical Statistics Faculty of Public Health, College of Medicine, University of Ibadan, Ibadan, Nigeria

**Keywords:** Neonatal mortality, Trends, Determinants, Nigeria

## Abstract

**Background:**

Nigeria’s efforts to reduce under-five mortality has been biased in favour of childhood mortality to the neglect of neonates and as such the literature is short of adequate information on the determinants of neonatal mortality. Whereas studies have shown that about half of infant deaths occur in the neonatal period. Knowledge of the determinants of neonatal mortality are essential for the design of intervention programes that will enhance neonatal survival. Therefore, this study was conducted to investigate the trends and factors associated with neonatal mortality in Nigeria.

**Methods:**

This was a retrospective analysis of the reproductive history data collected in the Nigeria Demographic and Health Surveys (NDHS) for 1990, 2003, 2008 and 2013. Neonatal mortality rates were estimated as the probability of dying before 28 completed days using synthetic cohort life table techniques. Univariate and multiple Cox proportional hazards regression models were used to explore the effects of selected maternal and bio-demographic variables on neonatal mortality. The Hazard Ratio (HR) and its 95% Confidence Interval (CI) were estimated to prioritize obtained significant factors.

**Results:**

Nigeria neonatal mortality rate stagnated at 41 per 1000 live births between 1990 and 2013. There were rural-urban and regional differences with more deaths occurring in rural areas and northern regions. In 1990, antenatal care (HR = 0.76; CI = 0.61-0.95), facility delivery (HR = 0.69; CI = 0.53-0.90) and births interval less than 24 months (HR = 1.67; CI = 1.41-1.98) were significantly associated with neonatal deaths. Factors identified from the 2013 data were antenatal care (HR = 0.76; CI = 0.61-0.95), birth interval less than 24 months (HR = 1.67; CI = 1.41-1.98), delivery at health facility (HR = 0.69; CI = 0.53-0.90), and small birth size (HR = 1.72; CI = 1.39-2.14).

**Conclusion:**

There was little improvement in neonatal survival in Nigeria between 1990 and 2013. Bio-demographic and health care related characteristics are significant determinants of neonatal survival. Family planning should be intensified while government should improve the quality of maternal and child health services to enhance the survival of neonates.

## Background

Many countries in South Asia and Sub-Saharan Africa including Nigeria still have high under-five mortality unlike some countries in East Asia, Pacific, Latin America, Carribean, and Central/Eastern Europe that have made substantial progress in its reduction [1]. Globally, it appears attention is focused on childhood survival more than neonates. Reports showed that between 2000 and 2010, the annual rate of reduction for neonatal mortality (2.1%) worldwide is lower than 2.9% recorded for under-five mortality with the proportion of under-five deaths in the neonatal period increasing from 37% in 1990 to 44% in 2013 [1,2]. It goes without saying that overall success in child survival is contingent on a corresponding decline in neonatal mortality. Unfortunately, 39% of neonatal deaths worldwide are in Sub-Saharan Africa [1]. Nigeria provided 6% of the global neonatal deaths in 2005 [3] while the country moved from the third to the second position in terms of the highest number of neonatal deaths in the world between 2000 and 2010 [2].

The Nigeria Demographic and Health Survey (NDHS) 2013 estimated its Neonatal Mortality Rate (NMR) as 37 per 1000 live births which constituted about 54% of infant mortality. The burden of neonatal mortality in Nigeria was higher than that of the African region as a whole in 2009 (36 per 1000) [4]. However, there have been some improvement in infant and under-five survival with the former reducing from 100 per 1000 live births in 2003 to 67 per 1000 in 2013 [5]. The rate of reduction recorded for neonatal mortality (53 per 1000 to 37 per 1000) was lower than that for infant and under-five. Several studies have provided useful insights into the determinants of under-five mortality, which were reported to differ in their effects across the age span 0- 5 years [6,7]. Such age variation in the effect of childhood mortality determinants informed the investigation of factors associated with neonatal mortality. Studies on determinants of neonatal mortality have received attention in Indonesia [8], Bangladesh [9], India and Ethiopia [10]. Many of these studies which were designed using the Mosley-Chen framework [11] have shown that neonatal mortality is affected by socio-economic and proximate factors. However, the majority of studies on neonatal mortality were conducted in Asian countries which have reported substantial progress in child survival compared to neonatal survival [8,9]. Findings from the Asian countries may not be applicable to Nigeria or any other countries in Africa due to differences in social, cultural and economic characteristics. Unfortunately, most of the few local studies on neonatal mortality in Nigeria were conducted in tertiary health facilities and have focused mainly on causes of death in children [12]. These studies have identified neonatal tetanus, birth asphyxia, prematurity, septicaemia and pneumonia as the commonly reported causes of death [13-18]. The major drawback in these studies is their selection bias which is common to health facility-based studies. Demographic and Health Survey (DHS) data represents a more reliable source for identifying the risk factors of childhood mortality, although limited in its ability to provide information on causes of death. The representative nature of DHS data offers a great advantage in identifying the modifiable factors associated with neonatal death and useful for designing prevention/intervention programes. This paper describes the trends in neonatal mortality in Nigeria as well as the influence of bio-demographic and maternal characteristics over two decades (1990-2013). Results from the study provide additional information that could be useful in planning intervention programes for neonatal survival in Nigeria and other low-income countries (especially in the West Africa sub-region) with similar demographic characteristics.

## Methods

### Setting

According to the 2006 population and housing census, Nigeria’s population was 140,431,790 with an estimated national growth rate of 3.2% per annum [19]. On 1^st^ November, 2011 during the commemoration of the accretion of the world population to seven billion, the United Nations Population Fund (UNFPA) put the population of Nigeria at 167 million, making it the sixth largest in the world after China, India, USA, Indonesia and Brazil. Nigeria is made up of 36 states and a Federal Capital Territory. It is grouped into six geo-political zones/regions: North West, North East, North Central, South East, South West and South- South. Nigeria’s current level of urbanization is about 45% but the country has one of the world’s highest urbanization growth rates estimated at 5.3% per year [20]. Fertility has remained high with a Total Fertility Rate (TFR) of 5.7 since 2003. The highest TFR was in the North West Zone (7.3) and lowest in the South West Zone (4.5). TFR also varies by location (highest in rural areas), education and wealth quintile. The health indices are characterised by wide regional disparities and generally better in the southern than the northern regions [21].

### Data sources

The children component of the data from the Nigeria Demographic and Health Survey (NDHS) for 1990, 2003, 2008 and 2013 were retrieved as the database for this study. The data sources, retrieval processes and other details have been described in a larger study on the trends and effects of changes in determinants of childhood mortality in Nigeria [22]. However, it suffices to state that the NDHS were based on nationally representative sample of women aged 15-49 years and men aged 15-59 years who were selected using a stratified two-stage cluster sampling technique. Data were collected on key reproductive health issues by trained field workers via structured interviewer administered questionnaires. A key component of the data collection is the maternity history where women were asked about their birth histories. Data from the birth history have been recoded into separate records for individual children listed by the mothers with data on date of birth, sex of the child, current age, age at death (for dead children), and relevant background characteristics. The data used for estimating neonatal mortality rates in this paper were based on all live births in the 5 years preceding data collection. For the regression models, multiple births were excluded because they are known to have excess mortality risks in infancy [23].

### Study variables

The main outcome variable is the risk of neonatal death. Neonatal death is defined as death before 28 completed days. Therefore, time to death was measured in days and infants who lived beyond 28 days were censored at that time for the purpose of survival analysis. The independent variables were grouped into two - background and maternity characteristics. The background characteristics were maternal education, marital status, sex of the child, residence and geo-political region, source of household drinking water and type of toilet facility. Maternity characteristics included antenatal care attendance, skilled attendance at delivery, Tetanus toxoide injection in pregnancy, place of delivery, mode of delivery, size of baby at birth, birth order, preceding birth interval and maternal age at child birth.

### Data analysis

NMR was estimated as the probability of dying before 28 completed days using life table techniques. NMR estimates were obtained for the period 1990, 2003, 2008 and 2013. Estimates were also obtained for rural/urban areas and the geo-political regions.

To explore the effects of the independent variables on neonatal mortality, univariate Cox proportional hazards regression models were fitted separately for 1990, 2003, 2008 and 2013 NDHS data sets. Subsequently, a set of multiple regression models were developed using the 2008 and 2013 data sets with a view to taking advantage of their very large sample sizes. Model IA included socio-economic related variables such as region, maternal education, rural-urban residence, source of drinking water and toilet facility. Model IB captured health care characteristics – antenatal care, skilled delivery attendance, place of delivery and mode of delivery, while Model IC included bio-demographic factors – birth order, birth interval, sex of baby, size of baby at birth and maternal age at child’s birth. Model II-2008 combined all the variables retained from model I to see how they might explain regional differentials in neonatal mortality. Model III and IV were replicas of model II but using the NDHS 2013 and 1990 data respectively. The purpose was to explore changes in the effects of the variables across the different time points. At each stage of the modelling process, backward elimination procedure was employed with probability of removal set at 0.15. Effects of covariates were expressed as Hazard Ratio (HR) with their 95% Confidence Interval (CI). A 95% CI that include unity (1.00) implied that the variable concerned has no statistically significant effect on the risk of neonatal death. The analyses were weighted and adjusted for complex sample design of the NDHS. Stata version 12 (Stata Corporation, College Station, TX, USA) was used for all analyses.

### Ethical considerations

The study received formal ethical approval (approved protocol number- UI/EC/12/0160) from the Institutional Review Committee of University of Ibadan/University College Hospital, Ibadan, Nigeria (NHREC/05/01/2008a). Permission to use the data was obtained from ORC Macro International, the agency responsible for the worldwide Demographic and Health Surveys. The latest two in the series of the NDHS (2013 and 2008) were approved by the Nigerian National Health Research Ethics Committee (assigned number NHREC/01/01/2007).

## Results

### Background characteristics

A total of 74,060 live births within the five years preceding the 1990 (7902), 2003 (6029), 2008 (28647) and 2013 (31482) NDHS were included in the analyses. The background characteristics of these births are summarized in Table 1. The highest proportion of live births was from the North West region throughout the period (1990-2013). About two-thirds were in the rural areas and approximately half of the children were born to mothers with no formal education while 15% and 32.7% were born to mothers with secondary or higher educational attainment in 1990 and 2013 respectively. Futhermore, almost all the children were born to mothers who were currently married or in a union (1990: 96.2%; 2003: 94.5%; 2008: 95.5%; 2013: 95.2%). Household access to improved water source reduced from 65.5% in 1990 to 28.1% in 2003 but later increased to 55.0% in 2013.

**Table 1:** **Percentage distribution of live births according to selected background characteristics, Nigeria, 1990 – 2013**

**Factors**	**1990 (n = 7633)**	**2003 (n = 5783)**	**2008 (n = 27685)**	**2013 (n = 30384)**
***Background characteristics***	**%**	**%**	**%**	**%**
**Region**				
North West	30.3	30.2	27.9	31.6
North East	9.7	24.8	23.0	20.9
North Central	14.4	16.8	17.6	14.6
South East	13.1	8.7	8.5	8.8
South South	9.9	9.3	11.5	11.9
South West	22.6	10.2	11.5	12.2
**Residence**				
Rural	65.2	65.0	73.5	67.3
Urban	34.8	35.0	26.5	32.7
**Maternal education**				
None	58.6	50.5	50.5	47.0
Primary	26.4	24.3	22.8	20.3
Secondary and higher	15.0	25.2	26.7	32.7
**Marital status**				
Currently married/in union	96.2	94.5	95.5	95.2
Not currently married	3.8	5.5	4.5	4.8
**Sex of baby**				
Male	49.5	49.1	49.1	50.7
Female	50.5	50.9	50.9	49.3
**Household water source**				
improved	65.5	28.1	47.9	55.0
not improved	26.8	71.9	52.1	45.0
**Household toilet facility**				
improved	72.3	71.3	63.2	48.5
not improved	27.7	28.7	36.8	51.5
***Maternity Characteristics***				
Antenatal care	57.9	60.5	49.6	58.1
Skilled delivery	37.9	37.4	34.8	34.9
Tetanus toxoide injection in pregnancy	54.1	53.7	51.5	60.2
**Mode of delivery**				
CS- delivery	2.4	1.7	1.5	1.9
Non-CS delivery	97.6	98.3	98.5	98.1
**Size of baby at birth**				
Large	31.7	42.4	47.0	43.6
Average	52.5	43.3	38.3	40.4
Small	15.8	14.3	14.7	16.0
**Place of delivery**				
Health facility	40.0	34.9	31.2	36.1
Home	60.0	64.7	67.3	63.9
**Birth order**				
1	18.1	20.7	19.2	19.9
2 and 3	31.6	31.7	32.9	32.2
4+	50.3	47.6	47.9	47.9
**Birth interval**				
first births	18.1	20.7	19.2	19.9
< =24 months	25.5	21.6	21.7	26.5
above 24 months	56.4	57.7	59.1	73.5
**Maternal age**				
<20 years	14.2	15.3	13.6	12.5
20 - 35 years	73.4	71.6	72.4	73.4
36 years and above	12.4	13.2	13.9	14.1

With respect to maternal health care utilisation, there was virtually no change in antenatal care and skilled delivery assistance over the years. Antenatal care (by a doctor or nurse/midwive) declined from 57.9% in 1990 to 49.6% in 2008 and increased to 58.1% in 2013 while skilled delivery assistance was reported for 37.9% and 34.9% of live births in 1990 and 2013 respectively. The proportion of babies delivered in a health facility gradually declined from 40.0% in 1990 to 36.1% in 2013.

The proportion of first or fourth order birth remain the same over time in Nigeria (Table 1). Similarly, there was no change in the birth intervals as about 20% of all birth had preceding birth interval less than 24 months while about one-third were born within 24-36 months after a previous birth. Maternal age at child birth also remains the same with majority in age group 20-35 years (1990: 73.4%; 2003:71.6%; 2008: 72.4%; 2013: 73.4%). The percentage of multiple births were 3.4%, 4.1%, 3.4% and 3.5% in 1990, 2003, 2008 and 2013 respectively.

### Trends in neonatal mortality

**Figure 1:**
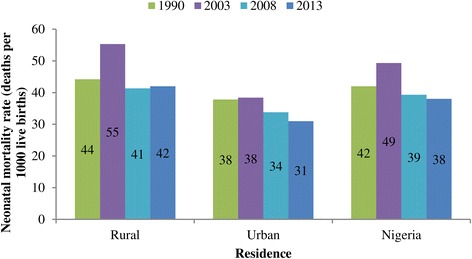
**Trends in NMR in rural and urban Nigeria, 1990-2013.**

**Figure 2:**
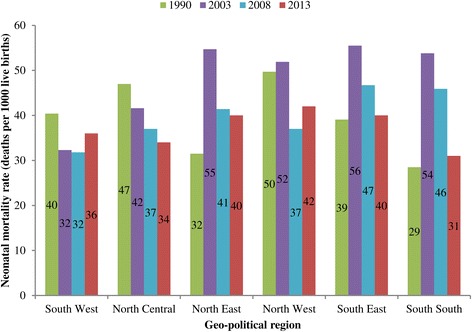
**Trends in NMR in Nigeria geo-political regions, 1990-2013.**

The Neonatal Mortality Rate (NMR) were 42, 49, 39, and 38 per 1000 live births for 1990, 2003, 2008, and 2013 respectively within five year preceding the surveys. Figure 1 shows the trend in NMR among live births in rural and urban areas in Nigeria. The peak NMR of 49 per 1000 was attained in 2003 survey. A similar pattern was observed in rural areas. However, a slightly different pattern was observed in the urban areas where the NMR was virtually the same between 1990 (38 per 1000) and 2008 (34 per 1000) but with some improvement in 2013 (31 per 1000). There are variations in the trends across the geo-political regions (see Figure 2). For instance, in the South West region, NMR declined between 1990 and 2003, stagnated at same level from 2003 to 2008 but slightly increased in 2013. A stepwise decline was recorded in the North Central throughout the study period while other regions had a rise in neonatal mortality between 1990 and 2003. The reverse was, however, the case between 2003 and 2008 when the regions exprienced varying degrees of decline with North East and North West having faster reductions. Figure 2 further shows that the South South region experienced the greatest decline in NMR between 2008 and 2013.

### Factor associated with neonatal mortality

The results of simple (univariate) Cox hazard regression for factors associated with neonatal mortality are presented for each survey (see Table 2). There were differences in the risk of neonatal deaths across the regions especially between the southern and northern regions. But these differences only attained statistical significance for North West versus South West in the 1990 survey (HR = 1.80; CI = 1.46-2.25). Also, the result showed that regional differentials in neonatal mortality had increased between 1990 and 2008. More neonates in the urban areas survived than their counterparts in rural areas throughout the study period (1990: HR = 0.76; CI = 0.65 – 0.90; 2003: HR = 0.59, CI = 0.49-0.72; 2013: HR = 0.77, CI = 0.65-0.91). The risk of neonatal death was also higher among births to women with no formal education compared to those with at least a secondary education. Likewise, neonates of women who were currently married or in a union had lower risk of death between 1990 (HR = 0.73, CI = 0.51-1.06) and 2008 (HR = 0.81, CI = 0.68-0.96). Male neonates had higher risks of death than female neonates, a pattern that was consistent during the period under review. Availability of an improved source of drinking water in the household was associated with survival advantage among neonates. Neonates whose mothers attended antenatal care, received skilled assistance at delivery and got at least a dose of tetanus toxoide injection were less likely to die. In contrast, those delivered by caesarean section have a significantly higher risk of neonatal death. Similarly, infants with small size at birth were at an increased risk - 1990 (HR = 1.82; CI = 1.46-2.27); 2013 (HR = 2.63; CI = 2.19-3.16). Births of order 1 and order 4 were also at higher risks of death in the neonatal period. Repeatedly, infants born with preceding birth interval less than 24 months were two times more likely to die as neonates compared to those born after 36 months. Births to mothers aged 20-35 years were less likely to suffer neonatal death compared to those of mothers younger than 20 years or older than 35 years.

**Table 2:** **Univariate Cox hazard regression analysis of neonatal mortality in Nigeria, 1990 - 2013**

**Factors**	**1990**		**2003**		**2008**		**2013**	
	**HR**	**95% CI**	**HR**	**95% CI**	**HR**	**95% CI**	**HR**	**95% CI**
***Socio-economic characteristics***								
**Region**								
North West	1.80	1.46 - 2.25*	2.13	1.51 - 3.02*	2.09	1.79 - 2.46*	1.22	0.95-1.57
North East	1.04	0.76 - 1.40	2.36	1.66 - 3.37*	2.01	1.70 - 2.36*	1.10	0.84-1.45
North Central	0.99	0.71 - 1.39	1.76	1.19 - 2.60*	1.59	1.34 - 1.89*	0.89	0.66-1.22
South East	1.29	0.98 - 1.69	0.87	0.55 - 1.38	1.79	1.48 - 2.18*	1.17	0.84-1.63
South South	0.88	0.63 - 1.23	1.82	1.19 - 2.76*	1.55	1.29 - 1.87*	0.94	0.68-1.32
South West (Ref)	1.00		1.00		1.00		1.00	
**Urban Residence**	0.76	0.65 - 0.90*	0.59	0.49 - 0.72*	0.64	0.58 - 0.71*	0.77	0.65-0.91*
**Maternal education**								
None (Ref)			1.00		1.00			1.00
Primary	0.77	0.64 - 0.92*	0.88	0.72 - 1.07	0.83	0.76 - 0.91*	1.09	0.90-1.32
Secondary and higher	0.57	0.43 - 0.75*	0.43	0.34 - 0.55*	0.61	0.55 - 0.68*	0.78	0.66-0.93*
**Marital status**								
Currently married/in union	0.73	0.51 - 1.06	0.98	0.69 - 1.38	0.81	0.68 - 0.96*	0.75	0.55-1.01
**Improved drinking water source**			0.99	0.84 - 1.17	0.75	0.70 - 0.81*	0.94	0.82-1.09
**Improved toilet facility**	0.84	0.71 - 0.99	0.77	0.65 - 0.91*	0.92	0.85 - 0.99*	0.88	0.76-1.02
***Healthcare characteristics***								
Antenatal care	0.56	0.48 - 0.66*	0.59	0.46 - 0.78*	0.74	0.65 - 0.83*	0.58	0.49-0.69*
Skilled delivery	0.76	0.64 - 0.89*	0.69	0.57 - 0.83*	0.69	0.64 - 0.75*	0.88	0.75-1.03
TT injection in pregnancy	0.50	0.43 - 0.59*	0.63	0.48 - 0.82*	0.71	0.63 - 0.80*	0.89	0.72-1.09
CS- delivery	1.46	0.91 - 2.35	0.35	0.16 - 0.77*	1.11	0.81 - 1.52	2.44	1.70-3.49*
Delivery at health facility	0.61	0.51 - 0.73*	0.54	0.44 - 0.66*	0.67	0.61 - 0.73*	0.92	0.79-1.08
***Bio-demographic factors***								
**Sex of baby**								
Male vs Female	1.19	1.02 - 1.39*	1.14	0.97 - 1.35	1.15	1.07 - 1.24*	1.33	1.15-1.54*
**Birth order**								
1	1.16	0.91 - 1.47	1.42	1.13 - 1.79*	1.09	0.98 - 1.22	1.59	1.31-1.95
2 and 3 (Ref)	1.00		1.00		1.00		1.00	
4+	1.28	1.06 - 1.55*	1.27	1.04 - 1.54*	1.19	1.09 - 1.30*	1.09	0.91-1.29
**Size of baby at birth**								
Large (Ref)	1.00		1.00		1.00		1.00	
Average	0.97	0.81 - 1.17	1.04	0.86 - 1.25	1.09	1.00 - 1.19	1.16	0.98-1.38
Small	1.82	1.46 - 2.27*	1.62	1.31 - 2.01*	1.79	1.63 - 1.98*	2.63	2.19-3.16*
**Birth interval**								
<24 months	1.64	1.38 - 1.95*	1.83	1.51 - 2.21*	1.92	1.76 - 2.09	2.08	1.75-2.47*
> = 24 months (Ref)	1.00		1.00		1.00		1.00	
**Maternal age**								
<20 years	1.20	0.98 - 1.47	1.36	1.10 - 1.68*	1.51	1.37 - 1.67*	1.54	1.27-1.85*
20 - 35 years (Ref)	1.00		1.00		1.00		1.00	
36 years and above	1.29	1.04 - 1.62*	1.25	0.99 - 1.57	1.21	1.09 - 1.35	1.22	0.99 – 1.50

*p<0.05 (statistically significant).

In order to control for confounding relationships between the variables, multivariable models were fitted for the hazards of neonatal death, with separate models for each survey. The results of these models are presented in Table 3. Model IA showed that the risks of neonatal death is higher among neonates in other regions compared to the South West. Conversely, urban residence, secondary/higher maternal education, marriage and availability of improved source of drinking water significantly reduces the risks of neonatal death. Results from Model IB for health care factors revealed that antenatal care, facility delivery and mode of delivery were important factors for neonatal survival. Antenatal care reduced the risk by 30% while neonates delivered by caesarean section are more than two times more likely to die (HR = 2.38, HR = 1.63-3.48). Model IC which assessed the effects of bio-demographic factors showed that male gender, high birth order (4 and above), small birth size, short birth interval (less than 24 months) and young maternal age (below 20 years) are risk factors for neonatal mortality.

**Table 3:** **Multiple Cox hazard regression analysis of neonatal mortality in Nigeria, 1990 - 2013**

**Factors**	**Model 1-2008**		**Model II-2008**		**Model III-2013**		**Model IV-1990**	
	**HR**	**95% CI**	**HR**	**95% CI**	**HR**	**95% CI**	**HR**	**95% CI**
***Socio-economic characteristics***								
**Region**	**Model 1A**							
North West	1.68	1.42 - 1.98*	1.36	1.01 - 1.82*	0.92	0.63-1.34	1.18	0.86 - 1.61
North East	1.60	1.35 - 1.90*	1.30	0.97 - 1.76	0.74	0.50-1.09	0.71	0.48 - 1.05
North Central	1.32	1.11 - 1.57*	1.15	0.86 - 1.24	0.69	0.47-1.05	0.80	0.55 - 1.18
South East	1.74	1.44 - 2.11*	1.41	1.01 - 1.95*	0.98	0.66-1.45	1.03	0.74 - 1.44
South South	1.38	1.14 - 1.67*	1.00	0.72 - 1.41	0.66	0.42-1.03	0.55	0.37 - 0.84*
South West (Ref)	1.00		1.00		1.00		1.00	
**Urban Residence**	0.77	0.70 - 0.85*	0.76	0.63 - 0.92*	0.73	0.57-0.93*	0.98	0.78 - 1.24
**Maternal education**								
None (Ref)	1.00		1.00		1.00		1.00	
Primary	-	-	1.03	0.85 - 1.24	1.28	1.01-1.63*	1.10	0.86 - 1.41
Secondary and higher	0.80	0.71 - 0.89*	1.03	0.82 - 1.30	0.98	0.72-1.35	0.90	0.59 - 1.38
**Marital status**								
Currently married/in union	0.75	0.63 - 0.89*	0.52	0.39 - 0.68*	0.59	0.38-0.91*	0.75	0.49 - 1.14
**Improved drinking water source**	0.89	0.82 - 0.96*	0.94	0.81 - 1.08	1.16	0.97-1.38	-	-
**Improved toilet facility**	-	-	-	-	-	-	-	-
***Healthcare characteristics***	**Model 1B**							
Antenatal care	0.70	0.61 - 0.81*	0.80	0.67 - 0.97*	0.59	0.47-0.74*	0.76	0.61 - 0.95*
Skilled delivery	-	-	-	-	-	-	-	-
TT injection in pregnancy	-	-	-	-	-	-	-	-
CS- delivery	2.38	1.63 - 3.48*	1.73	1.03 - 2.93*	3.57	2.19-5.82*	1.69	0.93 - 3.08
Delivery at health facility	0.87	0.73 - 1.02	1.05	0.85 - 1.29	1.17	0.88-1.54	0.69	0.53 - 0.90*
***Bio-demographic factors***	**Model 1 C**							
Male vs Female	1.15	1.05 - 1.25*	1.16	1.01 - 1.32*	1.35	1.14-1.60*	1.13	0.96 - 1.34
**Birth order**								
1	-		-	-	-	-	-	-
2 and 3 (Ref)	1.00		1.00		1.00		1.00	
4+	1.28	1.16 - 1.41*	1.11	0.94 - 1.30	1.03	0.83-1.27	1.22	0.98 - 1.55
**Size of baby at birth**								
Large (Ref)	1.00		1.00		1.00		1.00	
Average	1.09	0.99 - 1.20	-	-	-	-	-	-
Small	1.70	1.53 - 1.89*	1.36	1.15 - 1.60*	2.49	2.06-3.03*	1.72	1.39 - 2.14*
**Birth interval**								
< 24 months	1.90	1.75 - 2.07*	1.83	1.59 - 2.11*	2.02	1.69-2.41*	1.67	1.41 - 1.98*
>=24 months and above (Ref)	1.00		1.00		1.00		1.00	
**Maternal age**								
< 20 years	1.75	1.50 - 2.04*	1.48	1.13 - 1.93*	1.25	0.87-1.79	1.06	0.76 - 1.47
20 - 35 years (Ref)	1.00		1.00		1.00		1.00	
36 years and above	1.16	1.04 - 1.30*	1.54	1.31 - 1.80*	1.40	1.12-1.75*	1.23	0.98 - 1.55

*p<0.05 (statistically significant).

In Model II-2008 (Table 3), the variables that were significant from Model I A-C were entered into the model to control for possible confounding relationships. Despite the adjustment for other variables, there were still significant regional differential in neonatal mortality between the North West (HR = 1.36, CI: 1.01-1.82), South East (HR: 1.41, CI: 1.01-1.95) and the South West regions. Urban residence (HR: 0.76, CI: 0.63-0.92), marriage/union (HR: 0.52,CI: 0.39-0.68) and antenatal care (HR: 0.80, CI: 0.67-0.97) are protective against neonatal deaths. Infants who are males (HR: 1.16, CI: 1.01-1.32), of low birth weight (HR: 1.36, CI: 1.15-1.60), born within 24 months of a prior birth (HR: 1.83, CI: 1.59-2.11) are at a higher risk of neonatal death. Also, infants born to mothers aged less than 20 years or above 35 years have about 50% higher risk of death.

In order to explore the dynamics of the determinants of neonatal death, model II was re-fitted for the 2013 and 1990 NDHS data. The results showed that there was no significant regional differentials in 2013 whereas in 1990, differences existed between South South versus South West (HR: 0.55, CI: 0.37-0.84). From the 1990 survey, factors which significantly reduce the risk of neonatal mortality were antenatal care (HR: 0.76, CI: 0.61-0.95) and facility delivery (HR: 0.69, CI: 0.53-0.90). The risk factors were caesarean delivery (HR: 1.69, CI: 0.93-3.08), small birth size (HR: 1.72, CI: 1.39-2.14) and short birth interval (HR: 1.67, CI: 1.41-1.98). In the latest round of the NDHS (2013), antenatal care and facility delivery retained their protective effects while small birth size and short birth interval also remained as risk factors associated with neonatal mortality.

## Discussion

In this paper, we described the trend in neonatal mortality in Nigeria and explored the factors associated with neonatal death using the Nigeria Demographic and Health Survey collected in 1990, 2003, 2008 and 2013. This discussion starts with some comments on the distribution of selected background characteristics associated with neonatal mortality. While the percentages of births across regions are consistent, those of South West and North East were markedly different in 1990 compared to other years. The NDHS 1990 was originally designed to cover four regions that were in existence at the time (North East, North West, South East and South West). Following creation of new states in Nigeria, the regions have been re-structured into six. In the course of our analysis, we re-classified the regions in the 1990 data set in order to ensure consistency across all the surveys. Re-classification subsequently resulted in a little imbalance in distribution of births in 1990. Pattern of changes in variables such as access to improved water source and healthcare characteristics are largely related to increased population without improvement in basic infrastructure. This has been shown to be partly responsible for childhood mortality increase in Nigeria between 1990 and 2003 [22].

Our results showed that neonatal mortality has remained at very high levels with rural-urban and regional variation in trends over time. Doubling of the neonatal mortality in the South South region between 1990 and 2003 may be due to under-reporting of births and deaths in the 1990 survey especially in the North East and South East regions [24,25]. The fact that the South South region was re-classified out of the original South Eastern region in the 1990 survey may have also contributed to the observed pattern. Our findings on trends agree with previous results of trends in neonatal mortality in the world which showed that many countries in Sub-Saharan Africa have made little or no progress in neonatal survival [1,2]. Evidence on the historical patterns in neonatal mortality indicated that its decline usually lags behind that of under-five mortality [26,27]. In addition, progress in child survival is usually noticeable first between age 1 and 5 with attendant increase in the proportions dead before 28 completed days [28]. Given this established patterns, it is not suprising that very little progress had been recorded in neonatal survival in Nigeria between 1990 and 2013. Another related explanation for this result is the fact that most child survival interventions have targeted the post-neonatal period [29]. It is on record that several interventions for child survival have been implemented in Nigeria and other sub-Sahara Africa countries. The main challenge is sub-optimal coverage of these interventions which makes them not to have expected impacts. Cultural beliefs and practices are partly responsible for poor coverage of child survival interventions [30,31]. A recent analysis by Adedini et al. [32] has outlined cultural, physical and resource-related barriers as some of the factors affecting access to child healthcare services in Nigeria.

Regional differences in risks of neonatal deaths between the North East, North Central, South South and South West regions were reduced when other variables were controlled. This suggests that these maternal socio-economic and bio-demographic characteristics might largely be responsible for regional differentials in neonatal mortality. It is also noted that higher neonatal mortality risks still prevailed in North West and South East relative to the South West regions after controlling other variables. Regional differentials is a common phenomenon in the literature and has been partly attributed to differences in socio-economic, cultural/behavioural, nutritional and environmental charcateristics [33,34]. Nigeria Fertility behaviour and child care practices are deeply influenced by cultural norms, values and beliefs [30,35] which a cross sectional data such as the NDHS could not have adequately captured. Infants born to women in urban settings were found to have lower risk of neonatal death, and this urban advantage seems to have increased between 1990 and 2008. This is in contrast to findings in some other studies where the urban advantage either disappeared [10] or is reversed when other variables are controlled using multiple regression model [36]. Rural-urban differenecs have often been explained in terms of environmental factors and availability/utilization of health care services [37]. Urban women are more likely to go for antenatal care (due to better access to health care facilities) and as such abnormalities are more likely to be detected earlier and appropriate management instituted.

The influence of maternal education waned over time and also disappeared in the multivariable models. It means that all things being equal, whether an infant survives the first month of life may be independent of the mother’s educational attainment. This deviates from widely held views that maternal education remains important for children survival even when other variables are controlled [38]. However, maternal education is an index for socio-economic status which has been shown to be more important in the post-neonatal period [6]. Educated mothers are better able to make decisions on utilization of health care services [39] and adequate use of preventive and curative health services has greater effects on infants survival in the post-neonatal period [40].

Infants born to women in a marital union were less likely than those born out-of a union to suffer neonatal death. Meanwhile, a study in Ethiopia found that marital status was not significant in the multivariable model [10]. ‘Marital protection’ may be peculiar to the neonatal period in Nigeria context because culturally, Nigerian mothers and their babies enjoy a lot of familial and other social supports in the first month after birth. Women who are in a union have husbands who provide money for care and give psychological/social support to their wives.

Household environmental factors (source of potable drinking water and toilet facility) were not significant in the regression model. These factors have been shown to be important for infant and under-five mortality [41]. The fact that antenatal care remained significant in its protective effect underscores the importance of quality maternal care for neonatal survival. Quite a lot of child survival interventions such as health education/counselling, micronutrient supplementation, fetal monitoring, tetanus toxoide injection and others are provided during antenatal care. This result agrees with previous findings which showed that neonatal survival is intrinsically linked with proper maternal heath care services [42,43]. Other healthcare related variables such as skilled attendant at delivery and tetanus toxoide injection in pregnancy were eliminated during the modelling process. This perhaps also points to the fact that the effects of these variables were overtaken by that of antenatal care. Intuitively, the uptake of tetanus toxoide injection is consequent to antenatal care. In addition, only about one third of babies were delivered by skilled attendants while about half enjoyed antenatal care. Such imbalance in favour of antenatal care might have been responsible for the elimination of skilled delivery attendance from the models.

Bio-demographic factors such as sex of the baby, birth size, birth interval and maternal age at child’s birth were found to be important determinants of neonatal mortality. These results agreed with previous findings about the roles of these variables for child survival especially in the neonatal period. Male infants have higher mortality risks which has been attributed mostly to genetic factors in the absence of preferential care for female children [8,10]. There is no evidence for differential care/treatment between male and female children in Nigeria especially during infancy. Low birth weight infants are more likely to be victims of problems related to immaturity [13,15]. Our results further confirmed the danger associated with birth intervals less than 24 months. Infants born within 24 months of a previous birth are about two times more likely to suffer neonatal death [44]. This has been explained to be due to maternal depletion syndrome and other associated health problems. Infants of mothers aged below 20 years were also found to be at higher risks of neonatal death in Nigeria which implies that teenage pregnancy is risky not only for the mother but also for the infants. The lack of physical and physiological maturity required for good pregnancy outcomes is a major problem fuelling neonatal deaths of infants born to teenage mothers [6].

Certain limitations ecountered in the course of the analyses need to be borne in mind in interpreting the findings. Quite a number of neonatal deaths were reported to have occured on day 0. It is possible that some of these might have been stillbirths; unfortunately, not all the NDHS collected data with which still birth may be explored. Misreporting stillbirth as neonatal death might slightly affect the neonatal mortality rate, but this would not have affected the overall trend over time which is one of the main focus in the analyses. Data on birth history are subject to recall and displacement of events but evidence suggests that this might only bias the mortality rate by 5-7% [45]. Some important variables such as essential newborn practices and nutritional characteristics could not be included in the Cox model. This was because the data was either unavailable or was available for only surviving children. In addition, due to the cross sectional nature of the data, only association between the outcome and indepedent variables could be estabished. Causal relationship would require longitudinal study designs.

A major strength of this study is the fact that it leverages on nationally representative data collected via a consistent methodology between 1990 and 2013. In addition, this is the first known nationwide analysis that explores neonatal mortality in Nigeria, and as such could serve as benchmark and stimulus for further nationwide studies on the subject.

## Conclusion

This study shows that there was no much improvement in neonatal survival in Nigeria between 1990 and 2013. Rural-urban and regional differences exists and are partly explained by socio-economic, cultural, bio-demographic and maternal health related factors. The factors found associated with neonatal mortality are similar to those reported for other developing countries. In spite of these known factors, sufficient progress has not been recorded in neonatal survival in Nigeria. Antenatal care utilisation and bio-demographic factors such as birth intervals, maternal age and birth size are important determinants of neonatal mortality in Nigeria. Education of the girl child is one strategy to prolong the age at birth while family planning interventions holds the key to birth spacing. Improved coverage of antenatal and other maternal, newborn and child health care services will also guarantee the required progress in neonatal survival. Future studies need to provide insights on why progress in neonatal survival has been very slow. Research on innovative approaches to universal coverage of neonatal survival interventions are also desirable.
